# 2-Aminopyridine Analogs Inhibit Both Enzymes of the Glyoxylate Shunt in *Pseudomonas aeruginosa*

**DOI:** 10.3390/ijms21072490

**Published:** 2020-04-03

**Authors:** Alyssa C. McVey, Sean Bartlett, Mahmud Kajbaf, Annalisa Pellacani, Viviana Gatta, Päivi Tammela, David R. Spring, Martin Welch

**Affiliations:** 1 Department of Biochemistry, University of Cambridge, Cambridge CB2 1QW, UK; alyssacmcvey@gmail.com; 2 Department of Chemistry, University of Cambridge, Cambridge CB2 1EW, UK; sb956@cam.ac.uk (S.B.); spring@ch.cam.ac.uk (D.R.S.); 3 Aptuit LLC, an Evotec Company, 37135 VR Verona, Italy; mahmud.kajbaf@aptuit.com (M.K.); annalisa.pellacani@aptuit.com (A.P.); 4 Drug Research Program, Division of Pharmaceutical Biosciences, University of Helsinki, FI-00014 Helsinki, Finland; viviana.gatta@helsinki.fi (V.G.); paivi.tammela@helsinki.fi (P.T.)

**Keywords:** *Pseudomonas aeruginosa*, glyoxylate shunt, isocitrate lyase, malate synthase G, conditionally essential target, enzyme inhibitor, isothermal titration calorimetry

## Abstract

*Pseudomonas aeruginosa* is an opportunistic pathogen responsible for many hospital-acquired infections. *P. aeruginosa* can thrive in diverse infection scenarios by rewiring its central metabolism. An example of this is the production of biomass from C_2_ nutrient sources such as acetate via the glyoxylate shunt when glucose is not available. The glyoxylate shunt is comprised of two enzymes, isocitrate lyase (ICL) and malate synthase G (MS), and flux through the shunt is essential for the survival of the organism in mammalian systems. In this study, we characterized the mode of action and cytotoxicity of structural analogs of 2-aminopyridines, which have been identified by earlier work as being inhibitory to both shunt enzymes. Two of these analogs were able to inhibit ICL and MS in vitro and prevented growth of *P. aeruginosa* on acetate (indicating cell permeability). Moreover, the compounds exerted negligible cytotoxicity against three human cell lines and showed promising in vitro drug metabolism and safety profiles. Isothermal titration calorimetry was used to confirm binding of one of the analogs to ICL and MS, and the mode of enzyme inhibition was determined. Our data suggest that these 2-aminopyridine analogs have potential as anti-pseudomonal agents.

## 1. Introduction

Antibacterial resistance has intensified over the past several decades and threatens current standards of clinical care [1,2]. The majority of antibacterial drugs that are used today prevent or subvert just five essential cellular processes conserved across bacteria [3]. Although we have multiple antibiotic classes at our disposal, the rapid acquisition of resistance remains an ongoing issue [4]. Therefore, new antibacterial therapies are urgently needed. One of the pathogens where there is an unmet need for new antibacterial agents is *Pseudomonas aeruginosa*, an opportunistic pathogen renowned for its metabolic versatility and high-level intrinsic antibiotic resistance. Although traditionally cited as being “ubiquitous”, recent work has indicated that *P. aeruginosa* is primarily associated with human activity and the built environment [5]. 

*P. aeruginosa* encounters several metabolic challenges in vivo, particularly during infection scenarios, where nutrients are limited. When the primary source of carbon is derived from C_2_ molecules, the glyoxylate shunt is utilized to provide gluconeogenic precursors. The glyoxylate shunt is composed of two enzymes: Isocitrate lyase (ICL) and malate synthase (MS). ICL cleaves isocitrate to yield glyoxylate and succinate in a reversible reaction. This reaction is then followed by an irreversible condensation of glyoxylate and acetyl coenzyme A by MS, leading to formation of the gluconeogenic precursor, malate, and CoA [6,7,8].

ICL from *P. aeruginosa* (ICL*_Pa_*) is a tetramer, with each protomer comprised of 17 α-helices and 14 β-strands arranged as a TIM barrel-like core. The active site is located between β4 and β5 [9]. MS from *P. aeruginosa* (MS*_Pa_*) has also been characterized. The enzyme is a monomer comprised of an 8α/8β TIM barrel fold and an α-helical C-terminal domain, which borders the active site along with the TIM barrel [10].

Beyond their role in carbon fixation, ICL*_Pa_* and MS*_Pa_* have been implicated in virulence, persistence, and antibiotic resistance [11,12]. The glyoxylate shunt is conditionally essential for survival in mammalian systems, and a double deletion mutant (ΔICL ΔMS) of *P. aeruginosa* was found to be completely avirulent in a mouse pulmonary infection model [13]. Given their importance in pathogenicity and the fact that there are no human orthologues of the glyoxylate shunt enzymes, ICL*_Pa_* and MS*_Pa_* have become attractive targets for drug discovery efforts. Indeed, nature has already targeted the glyoxylate shunt as an antibacterial strategy. The human enzyme, Irg1, synthesizes the ICL-inhibitory metabolite, itaconate, during macrophage activation [14]. Several other ICL inhibitors have been identified, such as 3-bromopyruvate, 3-nitropropionate, and 2-vinyl-D-isocitrate [15,16,17]. However, these inhibitors display nonspecific hepatotoxicity, making them unsuitable as drug candidates [18]. 

Phenyl-diketo acid (PDKA) inhibitors of MS from *Mycobacterium tuberculosis* (MS*_Mt_*) have been developed through structure-based drug design. Their mechanism of action appears to be via chelation of the active site-bound Mg^2+^ by the 1-2-diol [19]. Further analysis of the structure-activity relationships indicated that these PDKAs adopt an unusual conformational pose once bound, which allows close contact between the PDKA aromatic ring and the carboxylic acid of a nearby aspartate side chain [20]. This pose permits the formation of an unusual face-on anion-π interaction. Optimization of the PDKA hits revealed that the potency of the inhibitors improved with increasing electrophilicity of the substituent at the ortho-position on the aromatic ring (2-F > 2-Cl > 2-Br > 2-Me > 2-H), resulting in stronger anion-π interactions [21]. 

Fahnoe and coworkers were the first to demonstrate the tractability of the glyoxylate shunt in *P. aeruginosa* as an antibacterial target using a combined chemical and genetic approach [13]. They identified a suite of eight compounds capable of preventing *P. aeruginosa* growth on acetate as a sole carbon source. Remarkably, these compounds also inhibited purified ICL*_Pa_* and MS*_Pa_* with IC_50_ values in the low micromolar range. However, the mechanism of inhibition, binding affinity, cytotoxicity profile, drug–drug interactions, metabolic clearance properties, and specificity of the compounds were not investigated further. 

**Figure 1:**
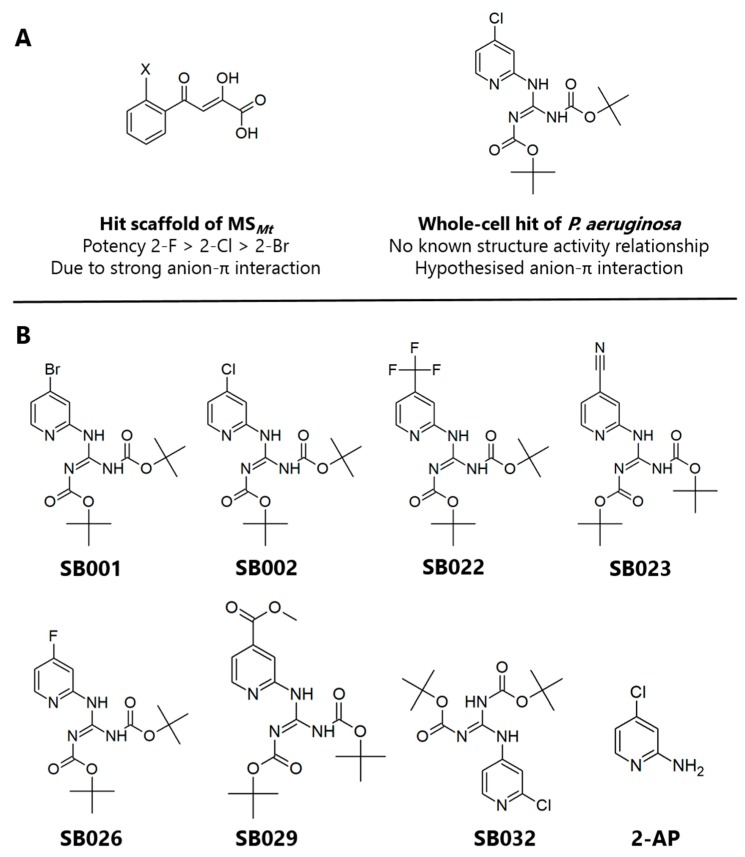
Structures of the 2-aminopyridine analogs characterized in this study. (**A**) Structures of glyoxylate shunt inhibitors reported in the literature with known or proposed anion-π interactions. (**B**) Compounds SB001-029 tested in the current work all shared a 2-aminopyridine core structure with tert-butyloxycarbonyl protecting groups, varying only in the substituent at the 4-position of the aromatic ring. SB032 and 2-amino-4-chloropyridine (2-AP) were synthesized as negative controls.

In the current study, we hypothesized that by combining the structural and electronic features of known inhibitors of the glyoxylate shunt enzymes, we might be able to improve their efficacy. Given its inhibitory activity against both ICL*_Pa_* and MS*_Pa_*, the 2-aminopyridine framework described by Fahnoe et al. [13] was considered a good starting point, especially given the π-acidity of the scaffold (Figure 1A). We, therefore, synthesized a suite of compounds, which all shared the core 2-aminopyridine structure but varied in the electronegativity of the substituents on the aromatic ring (Figure 1B). (Synthetic routes are outlined in the Supplementary Materials.) The targeted analogs were tested for their ability to inhibit purified ICL*_Pa_* and MS*_Pa_,* and the mechanism of inhibition. Crucially, we also further characterized the hits for cytotoxicity, in vitro drug clearance and cytochrome P450 inhibition, and protein binding. 

## 2. Results

### 2.1. Antibacterial Activity and Enzyme Inhibition

The 2-aminopyridine derivatives were tested for inhibition of purified ICL*_Pa_* and MS*_Pa_*. Complete inhibition of ICL*_Pa_* activity was observed with 75 µM SB002, compared with ca. 70% inhibition in the presence of 75 µM itaconate (ITA). The same concentration of SB002 also led to >90% inhibition of MSPa (Table 1). The other 2-aminopyridine derivatives showed less potent inhibitory activity. Dose-response curves confirmed the greater efficacy of SB002 against both enzymes (Figures S1, S2). 

The 2-aminopyridine derivatives were also assessed for their ability to prevent the growth of *P. aeruginosa* (strain PAO1) on rich medium (LB) and acetate as a sole carbon source. None of the compounds prevented bacterial growth in LB, except for SB026, which elicited slight growth inhibition (reflected by a diminution of ca. 23% in the final optical density achieved by the culture). This indicates that at the concentration tested (200 µM), the compounds are not generically toxic to *P. aeruginosa* (Table 1). However, when the same experiment was carried out with M9-acetate medium, SB002 and SB023 elicited essentially complete cessation of bacterial growth, with MIC values of 1.6 µM and 13.5 µM, respectively (Table 1 and Figure S3). SB001, SB026 and SB029 also led to slower growth, albeit to a lesser extent than SB002 and SB023 (Table 1). Interestingly, removal of the tert-butyloxycarbonyl (Boc) protecting groups from SB002 to yield 2-amino-4-chloropyridine abolished both the enzymatic and growth inhibitory activity of the compound.

### 2.2. Mode of Action

**Figure 2:**
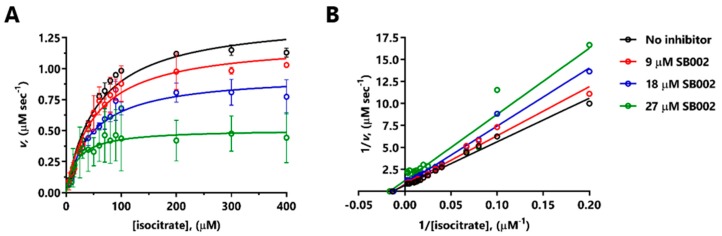
Mode of inhibition of ICL*_Pa_* by SB002. (**A**) Kinetic data obtained by titrating isocitrate in the presence of no additions (black symbols), 9 µM SB002 (red symbols), 18 µM SB002 (blue symbols), or 27 µM SB002 (green symbols). (**B**) The double-reciprocal plot showed that SB002 displayed mixed/noncompetitive inhibition of ICL.

SB002 was the most potent of the 2-aminopyridine derivatives tested, displaying even better inhibition of ICL*_Pa_* than itaconate (IC_50_ 13.3 µM for SB002 versus 24.9 µM for itaconate, Table 1). We, therefore, investigated the likely mode of action of SB002 further. SB002 displayed complex, concentration-dependent inhibitory kinetics with purified ICL*_Pa_*, affecting both the K_M_ and V_max_ of the enzyme (Figure 2A). When the data were transformed and presented as a double-reciprocal plot, a picture emerged of noncompetitive inhibition at the lower concentrations of the inhibitor, with evidence of uncompetitive inhibition at the higher concentration tested (Figure 2B). Indeed, the best fit model for the data (GraphPad Prism) was for uncompetitive inhibition, with an inhibition constant for binding to the enzyme-substrate (ES) complex, K_i_’, of 21.3 µM, and a negligible inhibition constant for binding to the free enzyme (K_i_ = 0 µM). The balance of evidence therefore indicates that SB002 likely binds to the ES complex in ICL*_Pa_*. 

**Figure 3:**
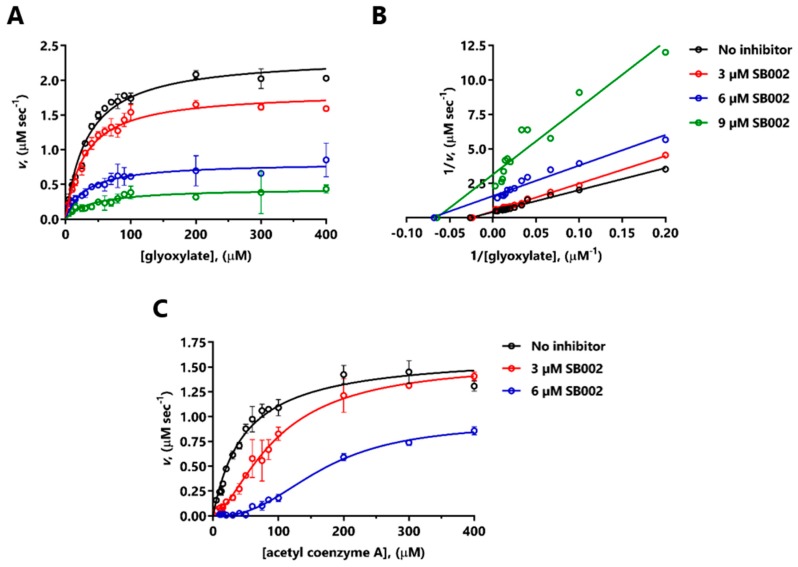
Mode of inhibition of MS*_Pa_* by SB002. The figure shows the kinetic data obtained in the presence of the indicated concentrations of SB002 and varying concentrations of glyoxylate at saturating (200 µM) acetyl-CoA concentration (**A**,**B**), or acetyl-CoA at saturating (200 µM) glyoxylate concentration (**C**). (**A**) and (**C**) show the untransformed data, whereas (**B**) shows the data transformed in the fashion of Lineweaver–Burk. Due to the change in the kinetic profile from Michaelis–Menten to sigmoidal in (**C**), we did not carry out a Lineweaver–Burk transformation.

SB002 elicited a large decrease in V_max_ of MS*_Pa_* for glyoxylate (Figure 3A). The corresponding double-reciprocal plot (Figure 3B) indicated uncompetitive inhibition at the lower inhibitor concentrations tested, and noncompetitive inhibition at the higher concentration of inhibitor. The resulting calculated inhibition constants were K_i_ = 4.5 µM (SB002 binding to the free enzyme) and K_i_’ = 3.9 µM (SB002 binding to the ES complex). MS*_Pa_* is known to exhibit ordered binding kinetics, with glyoxylate binding a necessary prelude to acetyl-CoA binding [10]. At a saturating concentration of glyoxylate, SB002 elicited a change in the kinetic profile from Michaelis–Menten (hyperbolic kinetics) to sigmoidal, with a large apparent increase in K_M_ with increasing inhibitor concentrations (Figure 3C). This is broadly consistent with a competitive mode of inhibition [22]. The shift to sigmoidal kinetics in the presence of SB002 was unexpected since MS*_Pa_* is a monomer in solution, and may be indicative of so-called “mnemonic” behavior [23].

### 2.3. Direct Binding of SB002 to ICL_Pa_ and MS_Pa_

**Figure 4:**
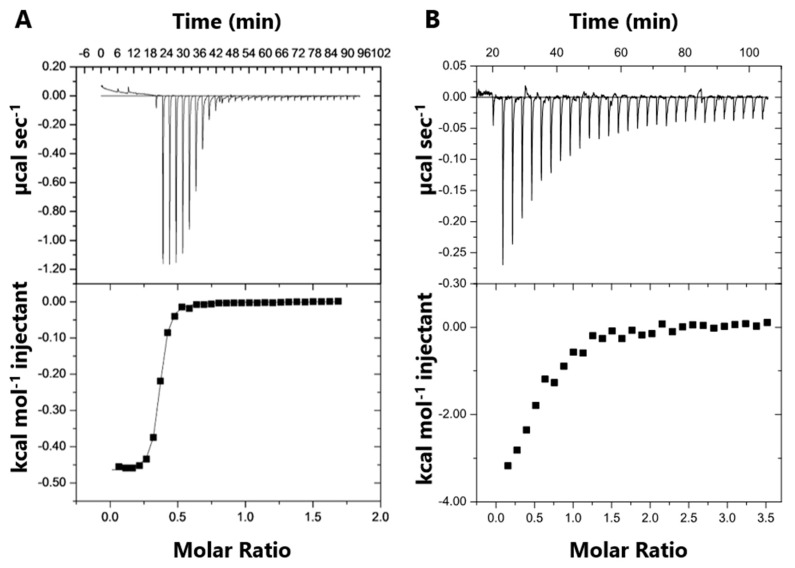
Isothermal titration calorimetry (ITC) data for SB002 binding to MS*_Pa_* and ICL*_Pa_*. The top panels illustrate the raw ITC data from 30 equal injections of either (**A**) MS*_Pa_* or (**B**) ICL*_Pa_*. Both interactions were exothermic. The bottom panels illustrate the respective binding isotherms obtained by plotting the integrated heat peaks against the molar ratio of protein to ligand.

SB002 binding to ICL*_Pa_* and MS*_Pa_* was measured using isothermal titration calorimetry (ITC). Purified MS*_Pa_* or ICL*_Pa_* was titrated into a calorimeter cell containing a fixed concentration of SB002 (Figure 4). This approach was necessary due to the limiting solubility of the ligand. SB002 became saturated with both proteins after 10 injections. The derived thermodynamic parameters are shown in Table 2. 

The number of binding sites (*n*) in the proteins was also calculated from the molar ratio of protein to ligand near to the inflection point of the binding isotherm (Figure 4). Because a reverse titration was performed, SB002 was in excess early in the experiment. If there is more than one binding site in the protein, then both sites will be occupied by the ligand and will contribute to ΔH (e.g., H_site 1_ +H_site 2_) [24]. Once enough protein has been added to interact with all of the ligands in the cell (after the inflection point in the binding isotherm), then ligand molecules occupying the weaker binding sites dissociate and instead occupy the higher-affinity site [25]. This yielded an *n* value of 0.53 for SB002 binding per monomeric unit of ICL*_Pa_*. We concluded that the binding stoichiometry was two molecules of SB002 per tetramer of ICL*_Pa_*. For MS*_Pa_*, we obtained an *n* value of 0.35 for SB002 binding per monomeric unit of MS*_Pa_*. This suggests that one molecule of SB002 binds for every three molecules of MS*_Pa_*. This suggests that MS*_Pa_* has the potential to form multimers. Consistent with this, we note that in Figure 3C, the binding kinetics are sigmoid, indicating possible higher-order structural transitions or protein aggregation upon SB002 binding. 

### 2.4. Cytotoxicity

The potential cytotoxicity of SB002 and SB023 was evaluated against three human cell lines: A-375 (from a malignant melanoma cell line), H27 (a foreskin-derived fibroblast cell line), and U937 (a lymphocyte cell line). Cytotoxicity against the A-375 and H27 cell lines was evaluated by quantitating ATP (as a proxy for cell viability) using a luminescence-based assay, whereas cytotoxicity against U937 was evaluated by measuring lactate dehydrogenase (LDH) release. In the concentration range required to elicit anti-pseudomonal activity (MIC 1.6 µM), SB002 was essentially nontoxic when tested against H27 cells (Table 3). Indeed, the therapeutic index (TI = 93) of SB002 against this cell line was large. However, SB002 was mildly cytotoxic against the U937 cell line, leading to a lower TI value (17). By contrast, and although less efficacious as an inhibitor of *P. aeruginosa* growth on acetate than SB002, compound SB023 was essentially completely noncytotoxic to U937 cells, resulting in a large TI (364) (Table 3). This large TI is encouraging; the FDA defines drugs with a narrow TI (i.e., less than a two-fold difference in the minimum toxic concentration and minimum effective concentration) as requiring clinical monitoring if approved [26]. 

### 2.5. Detoxification

Cytochrome P450 (CYP450) and its variants play an important role in the oxidative metabolism of almost 60% of drugs [27]. We, therefore, measured the in vitro metabolic clearance rates of SB002, SB023, and, as a negative control, “free amine SB002” (2-AP). The concentration of compounds used was chosen to represent the theoretical amounts of each test molecule free in the blood (i.e., not bound to plasma proteins). Each compound was incubated with human liver microsomes (HLM) and rat liver microsomes (RLM). Verapamil (metabolized by CYP450 3A4) and dextromethorphan (metabolized by CYP450 2D6) were also tested as positive controls. To predict hepatic clearance, the rate of parent compound depletion following incubation with the microsomes was plotted against the (log) percentage of analyte remaining (Figure S4). From this, we calculated the half-life of each compound and its intrinsic clearance rate (Cl_int_). Considering the body mass and blood flow of each organism, the data were also scaled to calculate the predicted hepatic clearance (Cl_h, int_), and the predicted in vivo hepatic clearance (Cl_h_) rates (see *Materials and Methods* for details). Interestingly, Cl_int_ for each compound displayed substantial interspecies variation; the values for Cl_int_ of the compounds incubated with HLM followed the order 2-AP > SB023 > SB002, whereas for RLM, the order was SB023 > SB002 > 2-AP (Table 4). As expected, the variation in microsomal clearance rates between compounds and species also carried through to the calculated Cl_h, int_ and Cl_h_ values. From the calculated hepatic clearance and blood flow, we also obtained an extraction ratio (*Materials and Methods*) for each compound. All tested compounds had an extraction ratio >0.70, indicating that the compounds are likely to be rapidly cleared from the blood by the liver and that their rate of clearance is heavily dependent on the rate of blood flow [28]. 

### 2.6. Drug-Drug Interactions

**Figure 5:**
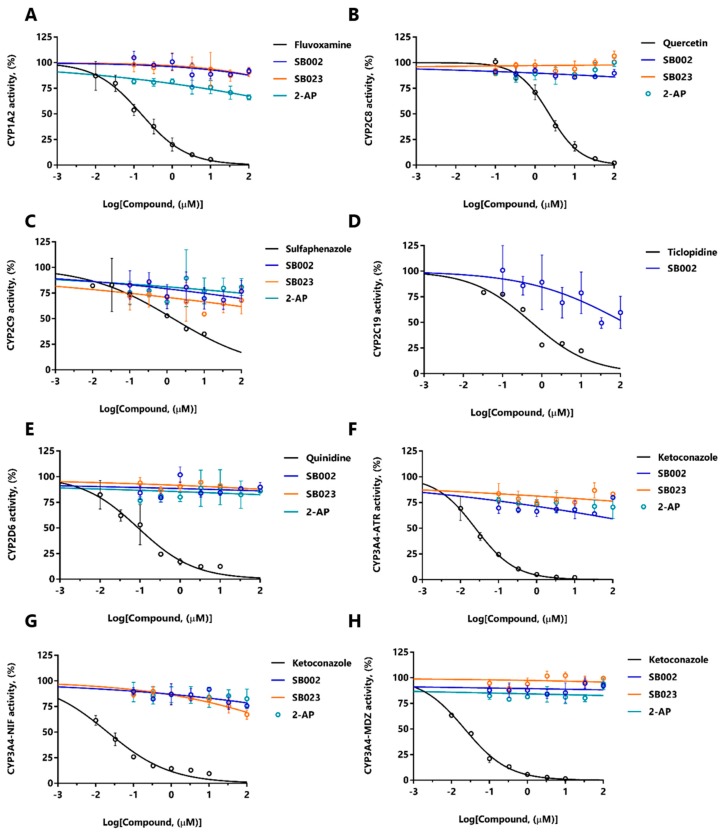
Dose-response inhibition of CYP450 isozymes. SB002 (blue), SB023 (orange), 2-amino-4-chloropyridine (teal), and relevant positive controls (black) were tested for their inhibition of: (**A**) CYP1A2 (positive control: Fluvoxamine), (**B**) CYP2C8 (positive control: Quercetin), (**C**) CYP2C9 (positive control: Sulfaphenazole), (**D**) CYP2C19 (positive control: Ticlopidine), (**E**) CYP2D6 (positive control: Quinidine), (**F**) CYP3A4 binding site 1 (ATR) (positive control: Ketoconazole), (**G**) CYP3A4 binding site 2 (NIF) (positive control: Ketoconazole), and (**H**) CYP3A4 binding site 3 (MDZ) (positive control: Ketoconazole).

The data in Table 4 suggest that SB002 and SB023 are CYP450 substrates. We, therefore, tested the possibility of potential drug–drug interactions arising from competition between these compounds and a suite of clinically relevant drugs known to also bind to the active site of CYP450s in HLMs. Such drug–drug interactions are important because they may lead to increased serum levels of the unmetabolized form [29]. To assess whether such drug–drug interactions occur, we used LC-MS/MS to examine the products of HLM-associated CYP450 action on a set of drugs that are known to be metabolized by different CYP450 variants. Phenacetin is metabolized by CYP450 variant 1A2, rosiglitazone by CYP450 variant 2C8, mephenytoin by CYP450 variant 2C19, bufuralol by CYP450 variant 2D6, atorvastatin, nifedipine, and midazolam by CYP450 variant 3A4, and diclofenac by CYP450 variant 2C9. By monitoring the ability of SB002, SB023, and 2-AP to interfere with the metabolism of drugs by these CYP450 variants, we were able to establish the possibility of drug–drug interactions. As positive controls, we also treated the HLMs with fluvoxamine (which is known to target CYP450 variant 1A2 and inhibit phenacetin turnover), quercetin (which targets CYP450 variant 2C8 and inhibits rosiglitazone turnover), sulfaphenazole (which targets CYP450 variant 2C9 and inhibits diclofenac turnover), ticlopidine (which targets CYP450 variant 2C19 and inhibits mephenytoin turnover), quinidine (which targets CYP450 variant 2D6 and inhibits Bufuralol turnover), and ketoconazole (which targets CYP450 variant 3A4 and inhibits atorvastatin, nifedipine, and midazolam turnover). The dose-response curves are shown in Figure 5. SB002, SB023, and 2-AP showed no major inhibitory drug–drug interactions over the concentration range tested, although SB002 did show some evidence of interacting with the turnover of mephenytoin (CYP450 variant C19, Figure 5D). By contrast, all positive controls inhibited CYP450s with IC_50_ values in the nanomolar to submicromolar range (Table S1). 

## 3. Discussion

In 2012, Fahnoe et al. reported that a pair of 2-aminopyridine (2-AP) derivatives, discovered following a high-throughput screen, could apparently inhibit both enzymes (ICL*_Pa_* and MS*_Pa_*) of the glyoxylate shunt [13]. This was a remarkable observation since ICL*_Pa_* and MS*_Pa_* exhibit very different structures and activities [9,10]. However, no detailed SAR was carried out, and, even for the hits, the mode of action, enzyme binding properties, cytotoxicity profiles, drug–drug interactions, and drug clearance properties of the compounds were not investigated further by Fahnoe et al. [13] In the current work, we rectified this and also carried out a limited SAR analysis of the 2-AP derivatives. 

The most potent inhibitor that we discovered (SB002, 4-chloro-2-AP) was identical to one of the 2-AP hits (referred to as compound 3) obtained by Fahnoe et al. in their high-throughput screen. However, in our hands, SB002 was a more potent inhibitor of MS*_Pa_* than it was of ICL*_Pa_* (IC_50_ = 4.5 µM and 13.3 µM, respectively), whereas Fahnoe et al. reported the opposite (IC_50_ = 0.17 µM for ICL*_Pa_* and 5.3 µM for MS*_Pa_*). The fact that the MIC of SB002 was lower than the IC_50_ for ICL*_Pa_* and MS*_Pa_* may indicate additional off-target effects or possibly differential partitioning of the compound into cells. SB002 lost activity as an enzyme inhibitor when the Boc protecting groups were removed and showed diminished activity when the chlorine atom at the 4-position on the pyridine ring was substituted with either bromine (SB001) or fluorine (SB026). Interestingly, a close analogue (SB029) of the most potent inhibitor identified by Fahnoe et al. (their compound 4, which is identical to SB029 except with a propionic acid substituent on the 2-AP ring instead of a methyl ester moiety) showed only weak activity in our experiments, indicating that inhibition may be exquisitely sensitive to even small structural variations. 

SB002 and SB023 could completely inhibit *P. aeruginosa* growth in M9-acetate, indicating that they are cell-permeable. The MIC for SB002 of 1.6 µM is comparable with the MIC reported previously by Fahnoe et al. for compound 3 (8 μg/mL, or 21.6 µM) [13]. Furthermore, the compounds had little or no impact on *P. aeruginosa* growth in LB, indicating that they are not generically toxic to the organism. It is notable that SB002 and SB023 are structurally dissimilar to the endogenous substrates of the glyoxylate shunt enzymes or with any of the known inhibitors of ICL. Crucially, SB002 and SB023 did not exhibit profound cytotoxicity towards any of the human cell lines we tested; although for the most part, they did exhibit a relatively narrow potential therapeutic index (TI < 100). This is not necessarily a major problem; there are several approved antibiotics or classes of antibiotics on the market with narrow TIs, including the aminoglycosides, vancomycin and polymyxin B [30]. SB002 and SB023 were metabolized by cytochrome P450 in human liver microsomes, with half-lives of 81 and 72 min, respectively (Table 4). These data suggested that both compounds are direct substrates of the CYP450 isozymes. However, neither compound prevented the microsomal CYP450 isozymes from metabolizing other drugs. 

Given that SB002 was (in our hands) the most potent inhibitor of both ICL*_Pa_* and MS*_Pa_*, we used enzyme kinetics to investigate its mode of action on each enzyme. SB002 displayed complex, concentration-dependent inhibition kinetics with MS*_Pa_*. At lower concentrations, SB002 elicited uncompetitive inhibition of MS*_Pa_* (indicative of inhibitor binding to the MS*_Pa_*-glyoxylate complex) but this changed to noncompetitive inhibition (inhibitor binding to the free MS*_Pa_* enzyme) at higher SB002 concentrations. The effects of SB002 on MS*_Pa_* kinetics with respect to acetyl coenzyme A were equally complex, although, overall, they were consistent with competitive inhibition. This was in line with our previous demonstration that the MS*_Pa_* acetyl coenzyme A binding site is a highly druggable binding pocket [10]. We speculate that SB002 could potentially interact with this pocket, thereby blocking the binding of acetyl coenzyme A at the protein’s surface. However, attempts to co-crystallize MS*_Pa_* with SB002 (± glyoxylate) or to obtain structures of the bound inhibitor-MS*_Pa_* complex through crystal soaking all failed. 

SB002 inhibition of ICL*_Pa_* was best described using an uncompetitive model, although the double-reciprocal plot data indicated more complex, “mixed” uncompetitive and noncompetitive inhibition (Figure 2B). Crousilles et al. reported that ICL*_Pa_* has a flexible loop between β4 and β5, which points away from the active site [9]. This loop contains the general catalytic base, Cys222. However, in the crystal structure of ICL from *M. tuberculosis,* the loop is positioned inwards. This closes off the active site and presumably brings Cys222 in close proximity to the substrate (isocitrate) for catalysis [9]. We speculate that when the substrate binds to ICL*_Pa,_* this leads to closure of the active cleft, and it seems plausible that SB002 binding to a nearby site somehow prevents this. Unfortunately, attempts to co-crystallize ICL*_Pa_* with SB002 failed to yield diffracting crystals. 

ITC revealed that SB002 binds to MS*_Pa_* with a greater affinity than to ICL*_Pa_*, which correlates well with the kinetic data. However, and as outlined above, Fahnoe et al. found that SB002 is a better inhibitor of ICL*_Pa_* [13]. The reason for this apparent discrepancy is not yet clear, although one possibility is that batch-to-batch variation in the enzyme preparations (i.e., the amount of integrally active enzyme present) might be responsible. We note that our preparations were “crystallization grade” enzymes, although we cannot exclude the possibility that some of the enzyme in each preparation could have been inactive.

In summary, we have shown that the 2-aminopyridines display the remarkable property of being able to inhibit two consecutive enzymes in a conditionally essential metabolic pathway, the glyoxylate shunt. This is all the more surprising given the very different structures and mode of action of each enzyme. Our top hit compounds, SB002 and SB023, displayed better potency than itaconate (a naturally occurring ICL inhibitor) and low cytotoxicity and drug–drug interactions. Current efforts are aimed at exploring the SAR of these compounds further and in establishing where they bind on the enzyme targets (through X-ray crystallography) with a view to employing structure-guided improvements in inhibitor design.

## 4. Materials and Methods 

### 4.1. Protein Purification of P. aeruginosa ICL and MS

ICL*_Pa_* and MS*_Pa_* were purified essentially as previously described [9,10].

### 4.2. Antibacterial Assays

Growth assays were carried out using clear, U-bottom, sterile 96-well plates (Thermo Fisher Scientific, Bishop’s Stortford, UK). *P. aeruginosa* PAO1 overnight cultures were diluted to a starting OD_600_ of 0.05 in LB medium or M9 medium supplemented with 0.5% acetate. The test compounds were added to the indicated final concentrations, and the plates were covered with gas-permeable adhesive seals (Scientific Laboratory Supplies, Nottingham, UK). Assay controls included 1% DMSO, a positive antibiotic control (ciprofloxacin, 1 µg/mL), and medium-only wells as a control for background absorbance. Plates were incubated in a FLUOstar® Omega microplate reader (BMG Labtech, Aylesbury, UK) for 18 h at 37 °C with shaking at 200 rpm, and automatic OD_600_ readings were recorded every 60 min. MICs were defined as the lowest concentration of test compound, which inhibited >75% of bacterial growth. 

### 4.3. Inhibitor Screening and Hit Confirmation

The enzymatic activity of purified ICL*_Pa_* and MS*_Pa_* was assayed essentially as previously described [9,10]. Compounds were added to the reaction mixtures (minus substrates) and incubated for 5 min at 37 °C. The enzyme concentration in the reaction mixtures was 170 nM for ICL and 25 nM for MS (based on the concentration of the monomer in each case). The DMSO solvent concentration did not exceed 1% *v/v*. The reactions were initiated by the addition of pH-adjusted substrates, and the initial rates were recorded as A_324 nm_ (for ICL*_Pa_*) and A_412 nm_ (for MS*_Pa_*) using a Biospectrometer (Eppendorf, Stevenage, UK). Substrate turnover was calculated from the molar extinction coefficient of the assay products: Glyoxylate-phenylhydrazone (16,800/M/cm) for ICL*_Pa_* and 2-nitro-5-thiobenzoic acid (14,150/M/cm) for MS*_Pa_*. 

### 4.4. Kinetic Mode of Action

The mode of action of SB002 on MS*_Pa_* and ICL*_Pa_* was determined by titrating the respective substrates of each enzyme in the presence of three fixed concentrations of SB002 (selected to lie around the measured IC_50_ value) and measuring the reaction rates as outlined in Section 4.3. Double-reciprocal plots and best-fit inhibition models (GraphPad Prism v. 7.04) were used to assess the mode of inhibition. Inhibition constants were calculated using GraphPad Prism v. 7.04 (GraphPad Software, Inc., La Jolla, CA, USA). Each data point represents the mean ± SD for three independent replicates.

### 4.5. Isothermal Titration Calorimetry

Protein samples were dialyzed (2 × 1 L) in a D-tube dialyzer (Merck, Watford, UK) for 3 h in 25 mM Tris-HCl, 100 mM NaCl, and 0.1 mM TCEP (pH 7.5). The protein samples were degassed and reverse-titrated into a calorimeter cell (VP-ITC, Malvern Panalytical, Malvern, UK) containing 11 µM of SB002 at 25 °C with 200 rpm stirring. The final protein concentration was 110 µM (MS*_Pa_*) or 202 µM (ICL*_Pa_*). The syringe and the cell both contained a final concentration of 0.1% DMSO. The injection parameters for MS*_Pa_* were: Reference power 15 µcal/sec, initial injection of 3 µL (3.6 sec per injection) with subsequent injections of 10 µL (12 sec per injection). Injections were spaced by 150 sec with a filter period of 2 sec. The injection parameters for ICL*_Pa_* were: Reference power 15 µcal/sec, initial injection of 3 µL (3.6 sec per injection) with subsequent injections of 8 µL (10 sec per injection). Injections were spaced by 170 sec with a filter period of 2 sec. Data were analyzed in NITPIC (NITPIC, Bethesda, MD, USA) [31] for baseline calculations and then fit for thermodynamic parameters using the OneSite model in Origin 7.0 software (Origin Software Solutions, Bristol, UK). Each titration was carried out in duplicate.

### 4.6. Cell Viability Assays

The number of viable cells based on ATP quantification was determined using the CellTiter-Glo® luminescent cell viability assay (Promega, Madison, WI, USA, lot no. 0000200446). Briefly, cells were seeded into white, flat-bottomed, sterile 96-well plates (PerkinElmer, Beaconsfield, UK) and incubated for 24 h. For A-375 cells, we used 10,000 cells/well, and for H27 cells, we used 7500 cells/well. Test compounds were diluted in assay medium (Glutamax DMEM with 5% FBS (Gibco)) and added to the plates in two-fold dilutions starting at 100 µM. Negative controls (0.5% DMSO) and positive controls (camptothecin, 1.6 µM for the A-375 cells and 50 µM for the H27 cells) were also included, and the plates were incubated for an additional 48 h (95% humidity, 5% CO_2_ at 37 °C). After incubation, the cells were washed with PBS. To each well, we then added 50 µL of assay medium plus 50 µL of CellTiter-Glo® reagent. The samples were then shaken for 2 min at 100 rpm at 25 °C, followed by a further 10 min at 25 °C to stabilize the luminescence signal. Luminescence was measured using a VarioSkan LUX multimode microplate reader (Thermo Fisher Scientific, Vantaa, Finland). 

The CytoTox 96® nonradioactive cytotoxicity assay (Promega, Madison, WI, USA) was performed to determine the number of cells affected by necrosis measuring LDH release. A U937 cell suspension (5 × 10^5^ cells/mL) was prepared, and 99.5 µL of this suspension were added to the wells of clear, sterile, 96-well, U-bottomed plates (Greiner, Dungannon, UK). The test compounds (0.5 µL of a 200 × concentrated stock) or 0.5% DMSO (control) were added to each well, and the plates were shaken for 1 min at 100 rpm, followed by 3.5 h at 37 °C with 5% CO_2_. At this point, CytoTox 96® 10× lysis solution was added to the wells reserved for total lysis. These samples represented the LDH released following complete cell lysis. After 30 min, 50 µL of supernatant from each test well and total lysis well was transferred to new 96-well plates (Thermo Fisher Scientific, Bishop’s Stortford, UK). Prethawed CytoTox 96® buffer was mixed with the CytoTox 96® substrate, and 50 µL were added into each well. The plates were incubated for 10 min in darkness at 25 °C. CytoTox 96® stop buffer (50 µL) was added, and the plates were incubated for a further 15 min. LDH release was quantified by measuring A_492 nm_ in a CLARIOstar® multimode microplate reader (BMG Labtech, Aylesbury, UK).

### 4.7. In Vitro Drug Metabolism

The metabolic stability of the test compounds was measured following incubation with liver microsomes from humans (BioIVT, lot no. IHG; West Sussex, UK) and rats (BD Gentest, lot no. 60614; BD Biosciences, Milan, Italy). Verapamil and dextromethorphan were used as positive controls. An automated incubation procedure was performed using the RSP Freedom Evo liquid dispensing and incubation system (TECAN, Milan, Italy). Five microliters of 50 µM test compound or positive control were added to 445 µL of microsomal suspension in 96 deep-well plates. The microsomal preparation comprised 0.56 mg/mL of pooled and homogenized liver microsomes suspended in 50 mM potassium phosphate buffer, pH 7.4. After 5 min at 37 °C, the reactions were initiated by adding 50 µL of prewarmed NADPH regenerating buffer to each sample. The regenerating buffer comprised a 2% NaHCO_3_ solution containing 1.7 mg NADP, 7.8 mg glucose-6-phosphate, and 6 units of glucose-6-phosphate dehydrogenase per mL. The samples were incubated at 37 °C, and 50 µL aliquots were withdrawn after 0, 3, 10, 15, 30, and 45 min and quenched in 150 µL acetonitrile. Aliquots (100 µL) of the quenched sample were mixed with 200 µL of internal standard (100 ng/mL Rolipram in water) prior to analysis on an API4000 Qtrap mass spectrometer (Applied Biosystems/MDS SCIEX, Monza, Italy) coupled to an HP1100 series HPLC system (Agilent Technologies, Milan, Italy) and a CTC-PAL auto-injector (CTC Analytics AG, Zwingen, Switzerland). The analytical column was a Synergi “Max-RP” reverse phase C12 with TMS endcapping (30 mm × 2 mm, 4 µm particle size) (Phenomenex, Inc., Bologna, Italy). The column temperature was maintained at 60 °C. The injected sample volume was 10 µL, and analytes were eluted at a flow rate of 800 µL/min in a gradient of eluent A (water with 0.1% formic acid) and eluent B (acetonitrile with 0.1% formic acid). The gradient conditions for elution were 5% B (0.00–0.20 min), 5–95% B (0.20–1.00 min), 95% B (1.00–1.30 min), 95–5% B (1.30–1.31 min), and 5% B (1.31–1.50 min). Analytes were quantified in multiple reaction-monitoring mode. The mass transitions used for positive controls and hit compounds are listed in Table S2. Peak areas were integrated using Integrator Software from Agilent (Agilent Technologies, Milan, Italy) and were divided by the peak areas of the internal standard. The percentage of parent compound remaining was calculated by normalizing the peak area ratio of the parent compound to that of the internal standard at time 0. The half-lives of compound metabolism were derived from the linear portion of the slope of ln percent parent compound vs time. Intrinsic clearance in liver microsomes was estimated from:

(1) $$Cl_{int}=k\times \left(\frac{V}{M}\right)$$

(2) $$Cl_{h,\;int}=\left(Cl_{int}\;\left(\times 1^{st}\;Scaling\;factor\right)\times 2^{nd}Scaling\;factor\right)\;$$

(3) $$Cl_{h}=\frac{\left(Q\times Cl_{h,\;int}\right)}{\left(Q+Cl_{h,\;int}\right)}.$$

Here, *k* is the rate of depletion of the parent compound (mL/min), *V* is the volume of the reaction mixture in mL, and *M* is the concentration of microsomal protein (in mg/mL). The amounts of protein in the incubations were scaled relative to the average liver- and body-mass of each species to obtain intrinsic hepatic clearance (*Cl_h, int_*, in mg/min/kg) (Table S3). These scaling factors were used along with the average hepatic blood flow (*Q*, in mL/min) of each species to obtain in vivo correlations (*Cl_h_*, in mL/min/kg) using the well-stirred model [32].

### 4.8. Cytochrome P450 Inhibition

The CYP450 inhibition was determined by measuring the reaction products of known CYP450 substrates (probes). Probe solutions were prepared by diluting stock solutions with buffer (100 mM Tris-HCl, pH 7.5, and 0.5 mM EDTA) to the final indicated concentrations (Figure 5), and adding 180 µL of pooled human liver microsomes (20 mg/mL) (BioIVT, West Sussex, UK). These probe solutions were dispensed (195 µL) into clear 96-well plates with low evaporation lids (Corning, Turin, Italy) and the plates were prewarmed to 37 °C in a thermomixer (Hamilton, Agrate Brianza, Italy). Next, 5 µL of the test compound or appropriate positive control (Table S4) were added to the wells and the plates were incubated for a further 5 min. Following this, the reactions were started by adding NADPH regenerating solution (50 µL, see previous Section 4.7) to each well. CYP450 mixtures containing phenacetin, rosiglitazone, mephenytoin, atorvastatin, and nifedipine probes were incubated for 10 min at 37 °C, whereas those with midazolam and diclofenac were incubated for 5 min at 37 °C. Aliquots (100 µL) from each reaction mixture were quenched in 200 µL acetonitrile, and the mixtures were sedimented at 3000 ×g for 10 min at 4 °C. The supernatants were diluted in water containing 5 ng/mL Rolipram as an internal standard for LC-MS/MS analysis. All incubations were carried out in triplicate.

Probe products were analyzed on an API4000 Qtrap mass spectrometer (Applied Biosystems/MDS SCIEX, Monza, Italy) coupled with an Acquity UPLC system (Waters, Milan, Italy) and a CTC-PAL auto-injector (CTC Analytics AG, Zwingen, Switzerland). For the CYP2C8 and CYP3A4 probe products, the analytical column was an Acquity BEH reverse phase C18 (50 × 2.1 mm, 1.7-µm particle size) (Waters, Milan, Italy). The column temperature was maintained at 60 °C. The injected sample volume was 10 µL, and analytes were eluted at a flow rate of 800 µL/min in a gradient of eluent A (water with 0.1% formic acid) and eluent B (acetonitrile with 0.1% formic acid). The gradient conditions for elution were 5% B (0.00–0.20 min), 5–95% B (0.20–1.50 min), 95% B (1.50–1.75 min), 95–5% B (1.75–1.80 min), and 5% B (1.80–2.00 min). For the CYP1A2, CYP2C9, CYP2C19, and CYP2D6 probe products, the analytical column was an Acquity BEH reverse-phase phenyl (50 × 2.1 mm, 1.7-µm particle size) (Waters, Milan, Italy). The column temperature was maintained at 45 °C. The injected sample volume was 10 µL, and analytes were eluted at a flow rate of 700 µL/min in a gradient of eluent A (water with 0.1% formic acid) and eluent B (acetonitrile with 0.1% formic acid). The gradient conditions for elution were 100% B (0.00–0.10 min), 100–20% B (0.10–2.30 min), 20% B (2.30–2.90 min), 20–100% B (2.90–3.00 min), and 100% B (3.00–3.80 min). The analytes were quantified in multiple reaction-monitoring mode. The mass transitions used for positive controls and hit compounds are listed in Table S5. Data were processed, and the percentage control activity was calculated based on a comparison between the ratio of the sample peak area and the peak area.

### 4.9. Data Analysis

All data fitting was performed with GraphPad Prism version 7.04 (GraphPad Software, Inc., La Jolla, CA, USA). The solid lines are the result of fitting the data to the equations denoted in relevant experimental sections. IC_50_ values were determined by a nonlinear fit using the equation for sigmoidal dose-response with variable slope. In all graphs, unless otherwise stated, the data points are the mean of experimental triplicates, and all error bars correspond to ± standard deviation. 

## Figures and Tables

**Table 1:** IC_50_ and MIC values for the 2-aminopyridine inhibitors of the glyoxylate shunt enzymes and *P. aeruginosa* PAO1 growth inhibition.

	IC_50_ (µM)		Enzyme Inhibition (%) at 75 µM		MIC (µM)		PAO1 Growth Inhibition ^a^ (%) at 200 µM	
Compound	MS*_Pa_*	ICL*_Pa_*	MS*_Pa_*	ICL*_Pa_*	LB	M9_ac_	LB	M9_ac_
SB001	>75	>75	16.2	6.1	>200	>200	0	48
SB002	4.5	13.3	94.5	100	>200	1.6	0	100
SB022	>75	>75	0	4.3	>200	>200	0	7
SB023	14.5	92.5	90.4	34.7	>200	13.5	0	97
SB026	59.3	54.5	55.1	62	>200	70.9	23	72
SB029	>75	64.7	0	55.9	>200	>78.9	6	45
SB032	70.6	91.8	48.7	43.8	n.d.	n.d.	n.d.	n.d.
ITA	-	24.9	-	72.6	-	-	-	-
2-AP	>75	>75	18.2	21	>200	>200	0	8

^a^ Growth inhibition refers to final optical density measurements of PAO1 culture after incubation with the compounds. ITA, itaconate; n.d., not determined.

**Table 2:** Thermodynamic parameters of SB002 binding to MS*_Pa_* and ICL*_Pa_*.

Thermodynamics	MS_Pa_	ICL_Pa_
Δ*G* (kcal/mol)	−10.31 ± 1.79×10^−3^	−7.98 ± 3.44×10^−1^
Δ*H* (kcal/mol)	−0.47 ± 1.79×10^−3^	−4.22 ± 3.44×10^−1^
−*T*Δ*S* (kcal/mol)	−9.84	−3.76
*K_a_* (M)	3.56 × 10^7^ ± 1.86 × 10^6^	7.18 × 10^5^ ± 1.49 × 10^5^
*K_d_* (nM)	28.60 ± 1.6	1,430 ± 0.30
*n* (binding sites)	0.35 ± 8.41 × 10^−4^	0.53 ± 3.25 × 10^−2^

**Table 3:** Cytotoxic potential of SB002 and SB023 against three human cell lines, A-375, H27, and U937. The therapeutic index compares the cytotoxicity results with results obtained from the dose-response inhibition of *P. aeruginosa* growth in M9 acetate medium (Figure S3) (IC_50_ cytotoxicity/IC_50_ *P. aeruginosa* growth inhibition).

	Cytotoxicity					
	A-375		H27		U937	
Compound	IC_50_ (µM)	Index	IC_50_ (µM)	Index	IC_50_ (µM)	Index
SB002	n.d.	n.d.	98.42	93.73	18.18	17.31
SB023	105.60	11.95	204.80	23.27	3,221	364.37

n.d.: not determined.

**Table 4:** Calculated in vitro and predicted in vivo clearance parameters of SB002, SB023, and 2-AP from human (HLM) and rat (RLM) liver microsomes.

	SB002		SB023		2-AP	
	HLM	RLM	HLM	RLM	HLM	RLM
**Cl_int_ (µL/min/mg)**	17.27	27.27	19.09	53.93	25.44	6.33
**Cl_h, int_ (mL/min/kg)**	23.31	62.99	25.75	124.58	34.33	14.63
**Cl_h_ (mL/min/kg)**	10.96	36.18	11.48	50.53	12.91	12.48
**T_1/2_ (min)**	80.50	50.99	72.85	25.78	54.65	219.58

## Supplementary Materials

Supplementary materials can be found at https://www.mdpi.com/1422-0067/21/7/2490/s1.

## Abbreviations

2-AP	2-amino-4-chloropyridine
ATR	Atorvastatin
Boc	Tert-butyloxycarbonyl protecting group
CYP	Cytochrome P450
DMEM	Dulbecco’s Modified Eagle Medium
DMSO	Dimethyl sulfoxide
FBS	Fetal bovine serum
ICL	Isocitrate lyase
LB	Luria –Bertani broth
LDH	Lactate dehydrogenase
MDZ	Midazolam
MS	Malate synthase G
NIF	Nifedipine
PDKA	Phenyl-diketo acid
SAR	Structure activity relationship
