# Supplementary material for: 2-Aminopyridine Analogs Inhibit Both Enzymes of the Glyoxylate Shunt in Pseudomonas aeruginosa
Source: Int J Mol Sci. 2020 Apr 3;21(7):2490. doi: 10.3390/ijms21072490 (PMC7177833; doi:10.3390/ijms21072490)
Supplement: Supplementary file 1 [file ijms-21-02490-s001.pdf]

Supplementary Material

2-aminopyridine analogs inhibit both enzymes of the glyoxylate shunt in *Pseudomonas aeruginosa*

Alyssa C. McVey[1], Sean Bartlett[2], Mahmud Kajbaf[3], Annalisa Pellacani[3], Viviana Gatta[4], Päivi Tammela[4], David R. Spring[2], and Martin Welch[1,*]

[1]Department of Biochemistry, University of Cambridge, Cambridge, United Kingdom

[2]Department of Chemistry, University of Cambridge, Cambridge, United Kingdom

[3] Aptuit LLC, Evotec, Verona, Italy

[4] Drug Research Program, Division of Pharmaceutical Biosciences, University of Helsinki, Helsinki, Finland

[*] **Correspondence:**
Dr Martin Welch
mw240@cam.ac.uk

Supplementary Figures

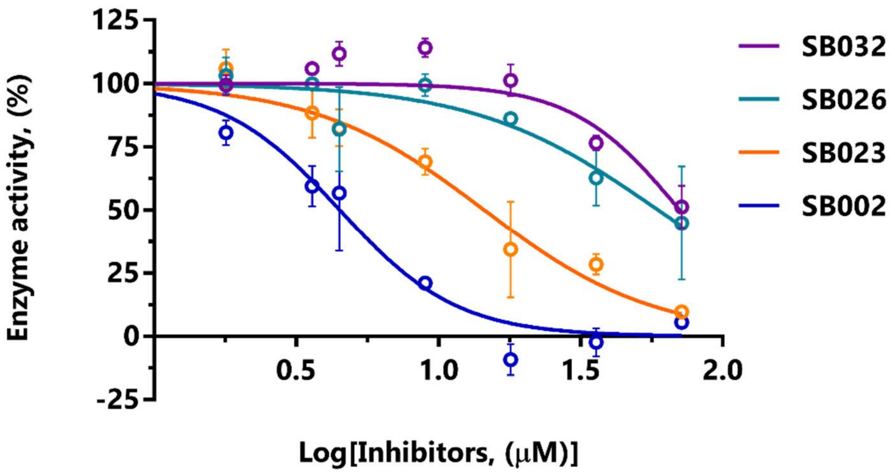

Figure S1. Dose-response inhibition of MS_{Pa}. SB002 (blue), SB023 (orange), SB026 (teal) and SB032 (violet) were tested at a range of concentrations (0-75 μM) and inhibited MS_{Pa} in a dose-dependent manner. Error bars represent biological triplicates with three technical replicates each.

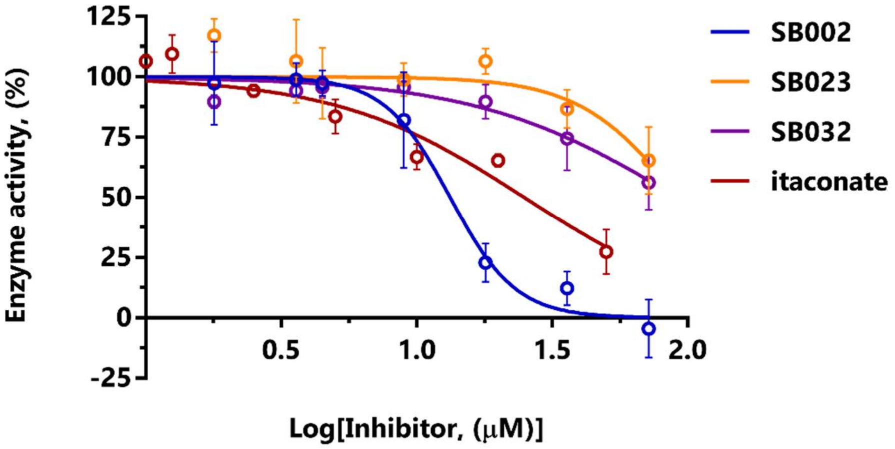

Figure S2. Dose-response inhibition of ICL*Pa*. SB002 (blue), SB023 (orange), SB032 (violet) and itaconate (maroon) were tested at different concentrations (0-75 μM; itaconate: 0-50 μM) and inhibited ICL*Pa* in a dose-dependent manner. Error bars represent biological triplicates with three technical replicates each.

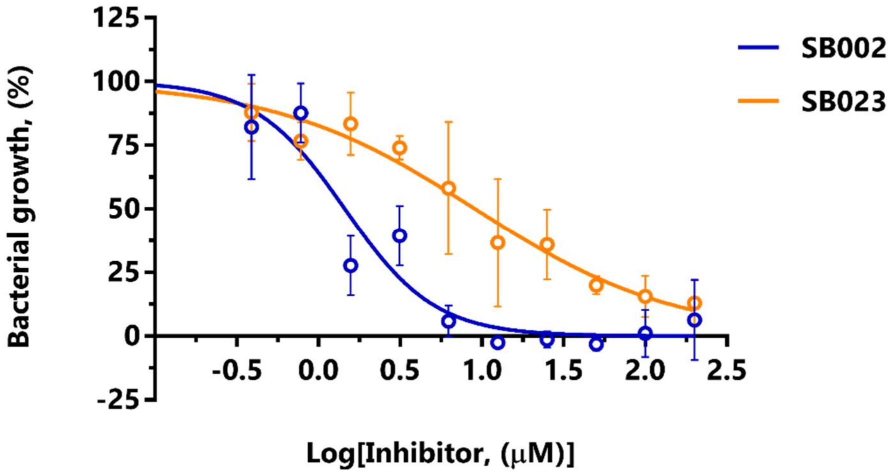

Figure S3. Dose-response inhibition of *P. aeruginosa* PAO1 growth by SB002 and SB023. SB002 (blue) and SB023 (orange) were added (0-200 μM) and inhibited PAO1 in M9 acetate in a dose-dependent manner. Error bars represent biological triplicates with three technical replicates each.

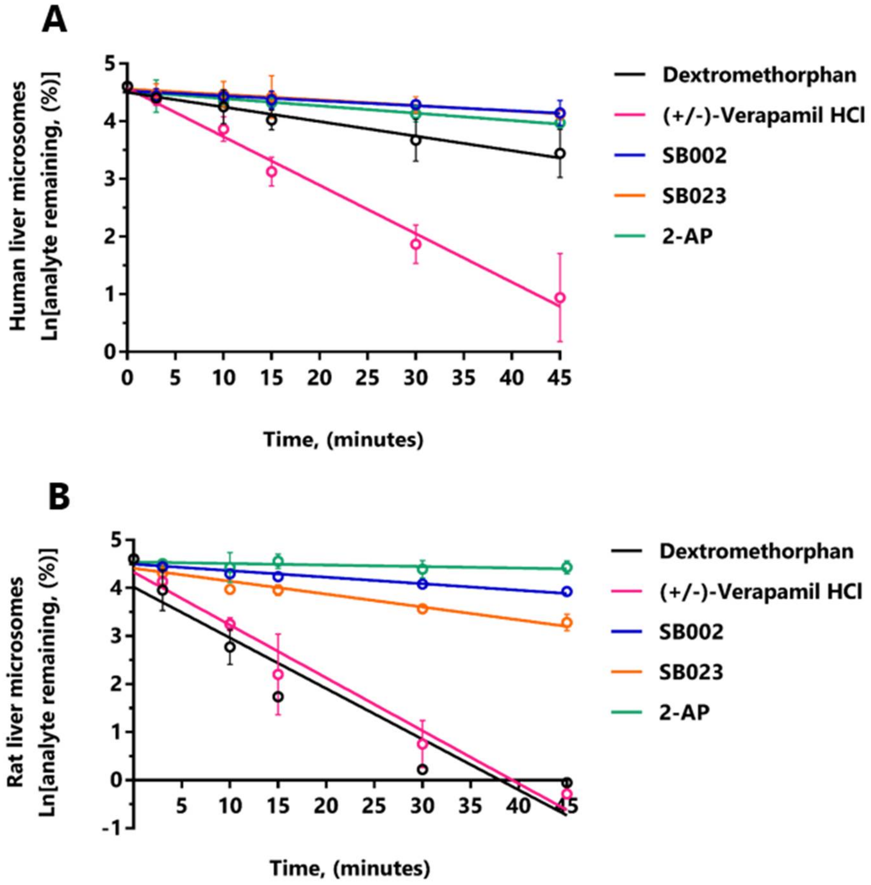

Figure S4. Metabolic clearance of SB002, SB023 and 2-AP in liver microsomes. All compounds were quantified by LC-MS/MS from 45-minute incubations with (**A**) human liver microsomes, and (**B**) rat liver microsomes. The % analyte remaining at each time point was calculated by dividing the LC-MS/MS peak area by the peak area of the internal standard (relative to the amount of analyte present at t = 0). Each value represents the mean of three independent experiments ± standard deviation.

Supplementary Tables

Table S1. Overview CYP450 inhibition. Parameters were determined from best nonlinear regressions of dose-response curves using GraphPad Prism version 7.04.

	IC$_{50}$ (μM)			
**CYP450**	**SB002**	**SB023**	**2-AP**	**Positive controls[a]**
1A2	>100	>100	>100	1.55 ± 1.16
2C8	>100	>100	>100	2.28 ± 1.14
2C9	>100	>100	>100	1.47 ± 1.81
2C19	126.20	n.d.	n.d.	0.54 ± 1.92
2D6	>100	>100	>100	0.09 ± 1.23
3A4-ATR	>100	>100	n.d.	0.03 ± 1.11
3A4-NIF	>100	>100	n.d.	0.02 ± 1.29
3A4- MDZ	>100	>100	>100	0.02 ± 1.16

[a] Positive controls; 1A2: fluvoxamine; 2C8: quercetin; 2C9: sulfaphenazole; 2C19: ticlopidine; 2D6: quinidine; 3A4-ATR, 3A4-NIF, and 3A4-MDZ: ketoconazole.

Table S2. Mass spectrometry ionization of hit compounds and controls.

**Analyte**	**Precursor ion (m/z)**	**Product ion (m/z)**	**Polarity**
SB002	371.0	259.0	Positive
SB023	362.2	145.1	Positive
2-AP	129.0	85.0	Positive
Rolipram (internal standard)	276.1	208.2	Positive
Verapamil (positive control)	455.1	165.1	Positive
Dextromethorphan (positive control)	272.1	171.1	Positive

Table S3. Species-specific body and liver masses, hepatic blood flows, and scaling factors used to calculate intrinsic clearance.

	**Liver microsomes species**	
	**Human**	**Rat**
**Scaling factor 1[a] (g)**	52.5	52.5
**Average liver weight (g)**	1800	11.0
**Average body weight (kg)**	70	0.25
**Scaling factor 2[b] (g/kg)**	25.7	44.0
**Blood flow (mL/min)**	20.7	85.0

[a] Aptuit's generic scaling factor for liver microsomal protein (mg) to liver (g).

[b] Scaling factor for liver mass (g) to body mass (kg) that is used to predict *in vivo* drug clearance.

Table S4. Substrate probes, probe products, and positive controls incubated with CYP450s.

CYP450	Substrate probes	Probe products	Positive controls
1A2	Phenacetin	Paracetamol	Fluvoxamine
2C8	Rosiglitazone	Par-hydroxyrosiglitazone	Quercetin
2C9	Diclofenac	4′-hydroxydiclofenac	Sulfaphenazole
2C19	S-Mephenytoin	4-hydroxymephenytoin	Ticlopidine
2D6	Bufuralol	1-hydroxybufuralol	Quinidine
3A4	Atorvastatin<br>Midazolam<br>Nifedipine	o-Hydroxyatorvastatin<br>1′-hydroxymidazolm<br>oxidized nifedipine	Ketoconazole

Table S5. Mass spectrometry ionization of probe products.

Analyte	Precursor ion (m/z)	Product ion (m/z)	Polarity
o-Hydroxyatorvastatin	575.10	440.40	Positive
Oxidized nifedipine	345.00	284.00	Positive
1′-Hydroxymidazolam	342.10	203.00	Positive
Paracetamol	152.00	110.20	Positive
Para-hydroxyrosiglitazone	374.00	151.00	Positive
4′-Hydroxydiclofenac	312.00	150.00	Positive
4-Hydroxymephenytoin	235.01	150.20	Positive
1-Hydroxybufuralol	278.40	186.00	Positive
Rolipram (internal standard)	276.10	208.20	Positive

Supplementary Methods

Method S1. Derivatisation of aminopyridines. Mercury(II) chloride (1.1 equivalent) is added to a solution of the aminopyridine (1.0 equiv.), 1,3-di-Boc-2-methylisothiourea (1.05 equiv.) and triethylamine (4.0 equiv. or 5.0 equiv. for hydrochloride salts) in CH_2Cl_2 (0.10 M) at 0 °C. The reaction mixture is stirred at 0 °C for 1 hour. The reaction mixture is allowed to warm to room temperature and stirred for 2-50 hours. The reaction mixture is charged with ethyl acetate (10.0 mL $mmol^{-1}$ amine) and filtered through a plug of Celite, which is washed through with ethyl acetate (1 × 25 mL). The combined organic phase is washed with water (1 × 20 mL) and brine (1 × 20 mL). The organic phase is dried ($MgSO_4$), filtered and concentrated under reduced pressure. The resultant material is chromatographed and/or recrystallised to afford the product.

Materials and methods. Reactions were carried out using standard Schlenk technique. Freshly distilled solvents were used under a nitrogen atmosphere in oven-dried glassware equipped with a magnetic stirring bar. Dichloromethane and hexanes were distilled from calcium hydride. All other reagents were used as received from commercial vendors. Masses of commercial reagents are absolute and do not account for reported percentage purity. Room temperature refers to the ambient laboratory temperature. Reactions were maintained at an external temperature of 0 °C using an ice–water bath and at -78 °C using an acetone–dry ice bath.

Reactions were monitored by thin layer chromatography. Thin-layer chromatography was performed using glass plates pre-coated with Merck silica gel 60 F_{254}. Visualisation was by the quenching of ultraviolet fluorescence (λ_{max} = 254 nm), or by staining with ceric ammonium molybdate solution (ammonium cerium sulfate, 2.0 g; ammonium heptamolybdate, 5.0 g; 98% sulphuric acid, 12 mL; water, 188 mL) or potassium permanganate solution (potassium permanganate, 4.0 g; sodium bicarbonate, 8.0 g; water, 200 mL). Retention factors (Rf) are reported to the nearest 0.01.

Flash chromatography[1] was carried out using a gradient of hexanes–ethyl acetate on Merck Kieselgel 9385 silica gel 60 or Sigma-Aldrich silica gel 60 (both 230-400 mesh) under a positive pressure of air. Automated chromatography was carried out using a Teledyne ISCO Combiflash Rf 200 chromatography system. Celite refers to AW Standard Super-Cel NF.

---

[1] Still, W. Clark.; Kahn, M.; Mitra, A. Rapid Chromatographic Technique for Preparative Separations with Moderate Resolution. *J. Org. Chem.* **1978**, *43*, 2923-2925.
